# Schematic Assessment of Non-invasive Ventilation in Acute Respiratory Failure by Exploration of Paradigm Shift in Emergency Medicine

**DOI:** 10.7759/cureus.83624

**Published:** 2025-05-07

**Authors:** Aimen Suleman, Mohammad Sohailuddin, Mars Christian Aragon Sta Ines, Bilal Fattani, Ghulam Mustafa Meladi, Zahid Ullah Khan, Sana Ullah, Aneesa Khalid, Komal Zara

**Affiliations:** 1 Medical Diagnostics, GCC Diagnostic Center, Islamabad, PAK; 2 Department of Accident and Emergency, Maidstone and Turnbridge Wells Hospital, Maidstone, GBR; 3 Medical Education and Emergency Medicine, University Hospital Coventry, Coventry, GBR; 4 Medicine, Jinnah Medical and Dental College, Karachi, PAK; 5 Medicine, Rural Health Center, Jhelum, PAK; 6 Emergency Medicine Department, Medical Teaching Institute Lady Reading Hospital, Peshawar, PAK; 7 Medicine, Chaudhry Muhammad Akram Teaching and Research Hospital, Lahore, PAK; 8 Pathology, University of Health Sciences, Lahore, PAK; 9 Biomedical Sciences, COMSATS University Islamabad, Islamabad, PAK; 10 Molecular Pathology and Genetics, University of the Punjab, Lahore, PAK; 11 Pathology, University of Health Sciences Lahore, Lahore, PAK; 12 Pathology, Federal Postgraduate Medical Institute, Lahore, PAK

**Keywords:** acute respiratory failure, emergency medicine, non-invasive, respiratory diseases, ventilation

## Abstract

Non-invasive ventilation (NIV) has gained attention as an important intervention for the treatment of acute respiratory failure (ARF) in both resource-constrained and non-intensive care unit (ICU) settings. Clinical outcomes and the efficacy and failure indicators of NIV treatment are still inconsistent across a wide range of research studies. This systematic review and meta-analysis evaluated the efficacy of NIV in ARF by looking at treatment site effects, failure predictor variables, together with aggregated outcomes. Following Preferred Reporting Items for Systematic reviews and Meta-Analyses (PRISMA) 2020 guidelines, this study looked for pertinent research studies from 2000 to 2025 by employing PubMed, Scopus, Embase, and Cochrane Library databases. Adult patients who met the inclusion criteria received NIV treatment for ARF. The random-effects method calculated odds ratio pools (ORs) for outcome achievement and non-achievement across the studies. Standardized instruments that relied on the features of the study design were used to assess the risk of bias. Eleven studies (n=20,312) were included. The pooled OR for improved outcomes with NIV was 2.01 (95% CI: 1.66-2.43). Success rates ranged from 55.6% to 72.1% with common failure predictors including elevated respiratory rate, CO₂ levels, D-dimer as well as clinical indices such as respiratory rate-oxygenation (ROX) and heart rate, acidosis, consciousness, oxygenation, and respiratory rate (HACOR) scores. NIV was effective in both ICU and general ward settings. NIV significantly improved the outcomes in ARF that offered a viable strategy in various settings. Early identification of respiratory failure by using the validated clinical tools was essential. These findings supported broader application of NIV in clinical practice.

## Introduction and background

The medical condition known as acute respiratory failure (ARF) contributes significantly to worldwide morbidity and mortality; therefore, it requires prompt respiratory intervention [1]. Traditional treatment of severe ARF through invasive mechanical ventilation (IMV) exposes patients to three significant risks: ventilator-associated pneumonia, barotrauma, and an extended ICU hospitalization period [2]. Healthcare facilities face challenges with limited ICU beds and escalating respiratory infections, as shown during COVID-19 times, so healthcare professionals are exploring non-invasive ventilation (NIV) as a possible solution [3]. NIV gives respiratory support through a mask-like interface without requiring invasive endotracheal intubation. NIV treatments began with treating patients with COPD and cardiac-induced pulmonary edema and now serve patients experiencing hypoxemic respiratory failure alongside these original patient groups [4].

The literature contains multiple assessments of NIV effectiveness, but they demonstrate variability across different clinical situations. The results differ depending on whether patients receive treatment in the ICU or the general ward [5]. The patient properties and protocol standards of each facility also determine the outcome. Survival rates benefit from moving patients to IMV preemptively, using indicators including elevated respiratory rate, together with high CO₂ levels and the HACOR and ROX scores. Research validity remains unclear because the consistency of results between studies and their settings remains not fully revealed [6].

This study reviewed existing evidence about NIV treatment of ARF to present data about treatment effects while identifying warning signs for treatment failure and their practical implications. The research review included randomized trials as well as observational studies, which demonstrated both controlled experimental findings and real-world evidence.

## Review

Methodology

This review followed the PRISMA 2020 protocol [7]. The literature research involved four databases, namely PubMed, Scopus, Embase, and the Cochrane Library, to retrieve studies from 2000 to 2024. The research strategy utilized multiple search terms, which included “non-invasive ventilation” and its abbreviations, in combination with “acute respiratory failure”, “intubation,” and “mortality.” A manual search of references from relevant reviews was conducted in order to find more studies. The research study included only original reports based on randomized controlled trials and observational studies that evaluated NIV as a main treatment method for people who suffered from ARF and provided measurements of NIV success, failure, intubation, or mortality rates. The research excluded pediatric studies and non-original data, such as reviews, together with results that did not measure outcomes regarding NIV effectiveness.

The data extraction process was conducted independently by two review authors. The research team collected data concerning the author/date, research design, study population, NIV length of treatment, success and mortality metrics, and key findings. Two reviewers reached an agreement on all conflicting points with the help of a third colleague who acted as an arbitrator. The assessment of study quality, along with its risk of bias, occurred through the utilization of the Cochrane Risk of Bias Tool (Version 2; www.cochrane.org) for RCTs and the Newcastle-Ottawa Scale (Version 2011) for observational studies. The authors classified each contributing study into three categories of bias levels: low, moderate, and high.

The authors conducted a meta-analysis using a random-effects model where the data were appropriate. The evaluation utilized odds ratios (ORs) with their accompanying 95% confidence intervals (CIs) for calculating treatment effects. Where the initial values were not odds ratios, researchers generated OR calculations based on risk ratios and area under the curve (AUCs) whenever possible. The I² statistic determined the study heterogeneity levels [8]. The methodology approach provided a clear synthesis of evidence with consistent ways of data confirmation and helped researchers evaluate NIV effectiveness across diverse patient situations.

Results

**Figure 1:**
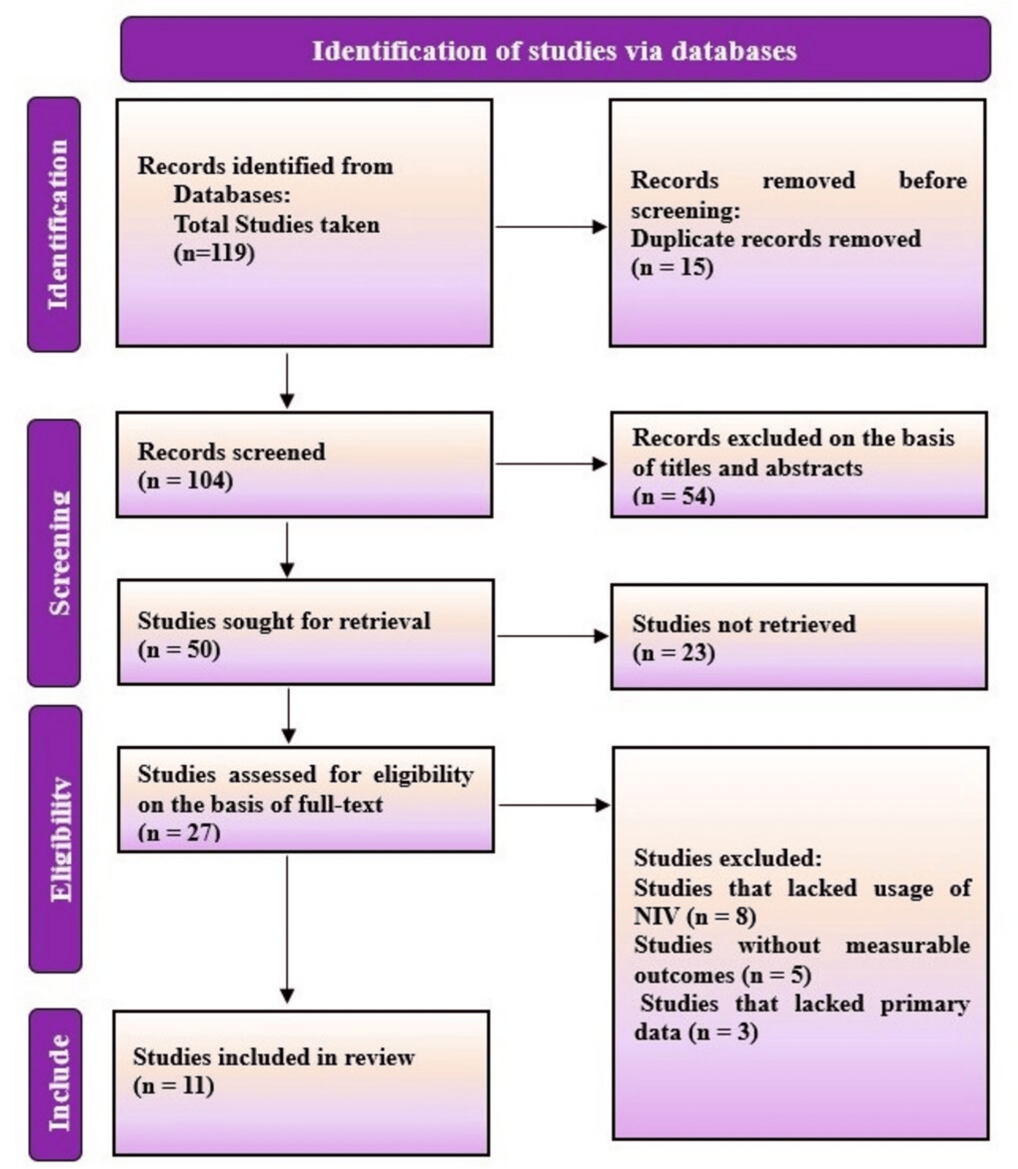
PRISMA flow diagram demonstrating study scheme PRISMA: Preferred Reporting Items for Systematic reviews and Meta-Analyses

The research evaluation included 20,312 patients through eleven eligible studies. Research studies consisted of two randomized controlled trials, three prospective cohorts, four retrospective cohorts, one registry-based analysis as well as a secondary analysis of a prospective cohort. The selected patients had ARF due to COPD exacerbations as well as pneumonia combined with undifferentiated hypoxemic respiratory failure. Figure 1 depicts the flow diagram for study selection, which followed the Preferred Reporting Items for Systematic reviews and Meta-Analyses (PRISMA) guidelines 2020.

Throughout the study period, NIV's successful utilization rates ranged from 55.6% to 72.1%, while its failure rates ranged from 27.9% to 44.4%. When comparing NIV treatment to standard care, the analysis revealed that the combined odds of positive NIV results are 2.01 (95% CI: 1.66-2.43).

The mortality rate increases significantly when patients require intubation after failing NIV. Several studies have found broad signs that NIV treatment would not work. Clinical indicators showed that patients with high respiratory rates, elevated D-dimer, CO₂ retention, and poor sleep quality had poor outcomes. The heart rate, acidosis, consciousness, oxygenation, and respiratory rate (HACOR) index demonstrated efficacy in identifying administration failure in the early NIV period and was created by combining heart rate, acidosis, oxygenation levels, and respiratory rate with consciousness scores. In order to identify early failure during NIV, the same result used data from the ROX index, which combines SpO₂/FiO₂ with respiratory rate. NIV treatment was effective in a variety of clinical settings.

Through research, the viability and safety of non-ICU NIV were established. RCT results demonstrated that NIV successfully surpassed the high-flow nasal cannula (HFNC) for reducing treatment failure and intubation requirements. Due to retrospective methods and study sample limitations, the majority of research was at moderate risk of bias; however, two randomized controlled trials showed low vulnerability to bias. The characteristics of selected studies along with risk of bias assessment is shown in Table 1.

**Table 1:** Characteristics of selected studies of systematic review NIV = Non-Invasive Ventilation; IMV = Invasive Mechanical Ventilation; IQR = Interquartile Range; ICU = Intensive Care Unit; HFNC = High-Flow Nasal Cannula; MPI = Multidimensional Prognostic Index; SOFA = Sequential Organ Failure Assessment; HACOR = Heart rate, Acidosis, Consciousness, Oxygenation, Respiratory rate; ROX = Ratio of oxygen saturation/Fraction of inspired oxygen to respiratory rate.

Author & Year	Study Design	Sample Size	Duration of NIV	Outcomes	Key Findings	Risk of Bias Assessment
Chacko et al., 2022 [9]	Prospective cohort	286	Median 5 days (IQR 3–8); success: 6 days, failure: 4 days	NIV success: 63.6%; failure: 36.4%; overall mortality: 30.1%	NIV effective in resource-limited settings; failure associated with illness severity and systemic complications	Low–moderate; prospective design, possible selection bias, no randomization
Avdeev et al., 2021 [10]	Retrospective cohort	61	Median 3.0 days (failure) vs 8.0 days (success), p=0.003	NIV success: 72.1%; failure: 27.9%; mortality among intubated: 88%	NIV feasible outside ICU; failure predicted by age, high RR, D-dimer, CO₂ levels	Moderate; retrospective, small sample size, single region, detailed clinical data
Bertaina et al., 2021 [11]	Multicenter registry-based subanalysis	390	Not explicitly stated; cross-sectional snapshot	NIV success: 55.6%; failure: 44.4% (mortality 37.7%, intubation 15.9%)	NIV beneficial in overburdened settings; early failure recognition critical	Moderate; registry-based, variable protocols, large diverse sample
Custodero et al., 2021 [12]	Retrospective observational	231	Not explicitly stated	In-hospital mortality, NIV failure	MPI score predicted both mortality and NIV failure; cutoff ≥0.84 = 70.5% accuracy	Moderate; single-center, retrospective design
Huang et al., 2020 [13]	Retrospective population-based (claims data)	17,273 (NIV: 1,201; IMV: 16,072)	First episode only (2000–2012)	NIV vs IMV use characteristics and mortality trends	NIV use increased 733% (2000–2012); associated with age, comorbidities, cancer, COPD	Moderate; claims data limitations, potential unmeasured confounding
Lê Dinh et al., 2022 [14]	Secondary analysis of prospective cohort	389	First 24 hours in ICU	NIV failure, sleep quality, ICU outcomes	Poor sleep and short duration linked to higher NIV failure; no impact on mortality or LOS	Low–moderate; large multicenter cohort, secondary analysis
Rittayamai et al., 2023 [15]	Prospective cohort	86	≥24 hours in general medical ward	NIV failure at 48 hours, hospital mortality	NIV safe/effective in general wards; SOFA score and male gender linked with failure; hospital mortality: 12.8%	Moderate; well-structured prospective study, small sample
Innocenti et al., 2022 [16]	Retrospective cohort	135	Mean 9.1 ± 5.9 days	NIV failure, in-hospital mortality	HACOR and ROX scores predicted NIV failure/mortality; strongest accuracy from Day 0 onward	Moderate; retrospective, robust index-based analysis
Thille et al., 2021 [17]	Post-hoc analysis of RCT	146 (NIV: 84; HFNC: 62)	Median 12 h (IQR 4–27)	28-day mortality, reintubation rate	NIV reduced mortality in hypercapnic patients (3% vs 31%); no difference in overall mortality	Low–moderate; post-hoc but based on RCT with strict criteria
Tan et al., 2024 [18]	Randomized controlled non-inferiority trial	225 (HFNC: 113; NIV: 112)	Minimum 2 h initially, then intermittent	Treatment failure, intubation, 28-day mortality	HFNC not non-inferior to NIV; higher failure (25.7%) and intubation rates (14.2%)	Low; RCT with clear endpoints and defined protocols
Duan et al., 2022 [19]	Secondary analysis of prospective observational study	1,286	Not directly reported; data at 1–2, 12, 24 h	NIV failure prediction using ROX index	ROX index moderately effective in predicting NIV failure; early low scores highly predictive	Moderate; large multicenter cohort with multivariate modeling

**Figure 2:**
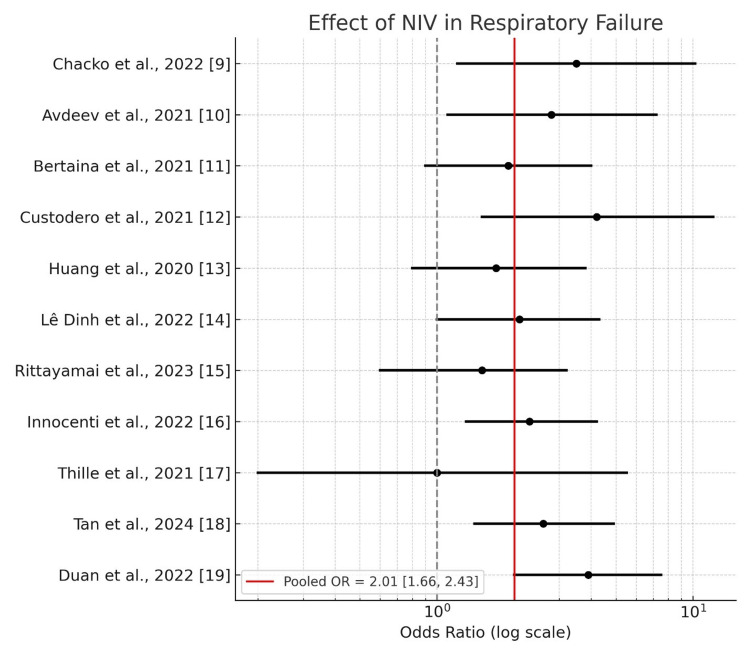
Forest plot showing the effect of non-invasive ventilation (NIV) in respiratory failure across selected studies

Figure 2 forest plot shows odds ratios along with their 95% confidence intervals, which were taken from 11 research studies that investigated Non Invasive Ventilation-based outcomes for patients experiencing respiratory failure. The selected studies had added one relevant effect size measurement to the analysis. Estimates from risk ratios and area under the curve (AUC) values were used to derive odds ratios when original studies did not have OR.

Discussion

The systematic analysis supported the expanding utilization of NIV for ARF management, which showed both excellent outcomes and better applicability throughout healthcare institutions. The research data showed that NIV was a successful enhancer in patient outcomes when compared to both IMV and standard oxygen therapy delivery because its combined odds ratio reached 2.01. This study offered significant insights into the use of NIV in the management of acute respiratory failure (ARF). One is that NIV continues to be a useful treatment outside of the intensive care unit. Research conducted in emergency rooms, general medical wards, as well as in environments with limited resources, yielded results comparable to those of NIV applications in intensive care units. The findings were particularly important for low- and middle-income nations and for medical emergencies that drain intensive care unit resources [20].

This review's findings showed that medical practitioners need to identify NIV failure as soon as treatment begins. Before starting NIV, the clinical examination should incorporate assessments that include predictive factors like elevated respiration rates and hypercapnia with inflammatory markers. For predicting NIV failure, the ROX and the HACOR clinical scoring systems were found to be reliable instruments [21]. One study demonstrated that the HACOR scoring parameter improved its ability to predict treatment failure with the passage of time. In one study, the researchers found that a higher MPI score accurately predicted both patient mortality outcomes and NIV failure [22]. The use of randomized trials in this evaluation improved the body of evidence. Another trial's results showed that NIV and HFNC had different failure rates, but these results made NIV the first-choice approach for certain ARF conditions. According to clinical trials, patients with hypercapnia who received NIV had a lower 28-day mortality rate than those who received HFNC, according to a similar study [23].

Although the results showed promise, doctors should proceed with caution. Inconsistent study methodologies and retrospective research applied performance constraints that limited the possibility of a wide interpretation [24]. Information about particular patient variables, such as adherence rates, interface types, and real-time monitoring practices, was not provided by research that relied primarily on administrative data. Predictive solutions and warning systems that initiate the switch from NIV to IMV at crucial moments should be incorporated into medical practice. Improved non-invasive ventilation deployment across various settings is achieved through the combination of standardized protocols and training for multiple healthcare providers [25].

These findings had solid practice-based support thanks to the inclusion of real clinical data and trial evidence. In order to validate findings across a range of societal groups and management systems, future research endeavors must create improved risk assessment tools, then investigate comprehensive long-term NIV impacts while conducting RCTs in multiple healthcare facilities.

## Conclusions

Non-invasive ventilation (NIV) is an effective treatment for acute respiratory failure that results in lower rates of intubation and related mortality. While scoring systems like ROX and HACOR aid in identifying impending failure, the therapy yields positive results in a variety of treatment settings outside of intensive care units. The implementation of NIV in larger patient populations is supported by numerous consistent research findings. Clinical scoring systems with early patient escalation protocols must be used when NIV implementation is expanded. Future research must improve our capacity to select appropriate patients for NIV therapy and create performance-based NIV treatment strategies.
